# P-714. Unequal Burdens: Gender, Geography, and Risk Factors Driving HIV and STI Trends in the U.S.—A GBD 2021-Based Systematic Analysis

**DOI:** 10.1093/ofid/ofaf695.926

**Published:** 2026-01-11

**Authors:** Sameer Kumar Majety, Anandalakshmi Ponnaluri, Hemanth Kamadi, Komuroju Pooja Mrinmai, Srinivasa Chakradhar Earni, V I nesh Seelam

**Affiliations:** School of Medicine, Xiamen University., Kakinada, Andhra Pradesh, India; School of Medicine, Arizona University, Phoenix, Arizona; School of Medicine, Xiamen University., Kakinada, Andhra Pradesh, India; Dr Patnam Mahender Reddy Institute of Medical Sciences, Hyderabad, Telangana, India; International Higher School of Medicine, Krygyzstan., Hyderabad, Telangana, India; School of Medicine, Xiamen University., Kakinada, Andhra Pradesh, India

## Abstract

**Background:**

Sexually transmitted infections (STIs), including HIV, remain a major public health concern globally and in the U.S. Beyond HIV, infections such as chlamydia, gonorrhea, syphilis, trichomoniasis, and genital herpes show marked variation across states and sexes.

**Figure 1:**
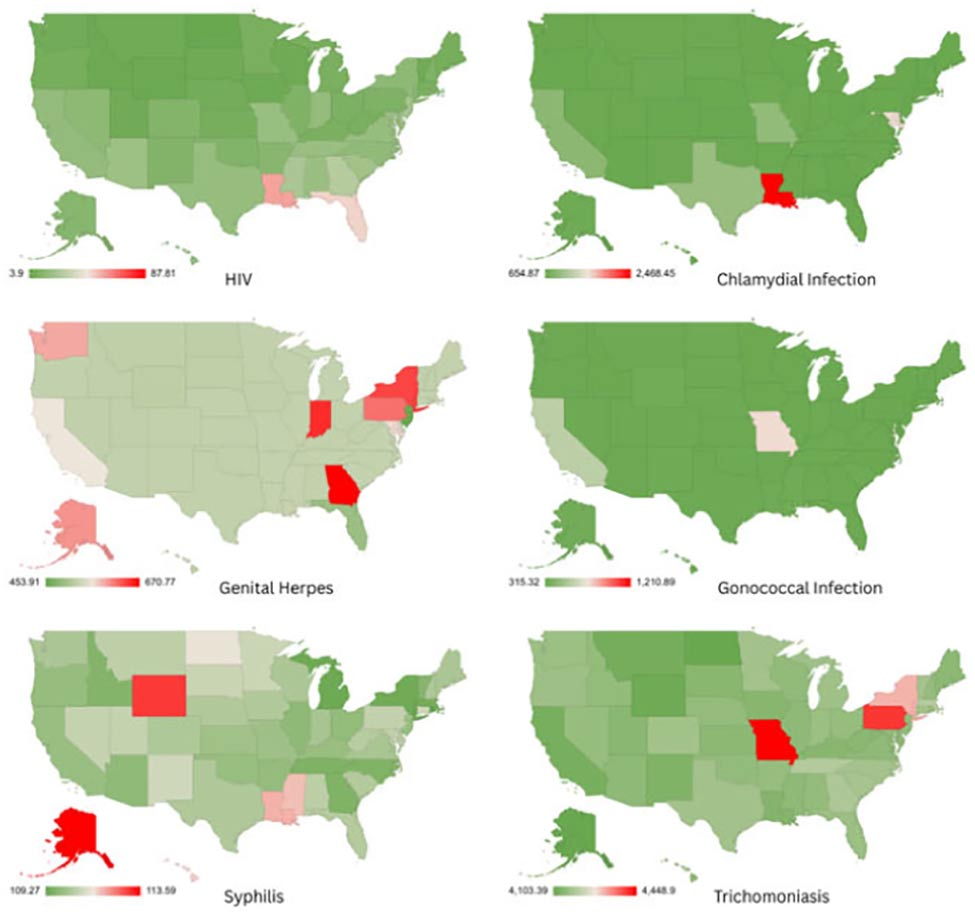
Choropleth maps depicting state-wise distribution of HIV and STI incidence rates, highlighting regional extremes across the United States.

**Figure 2:**
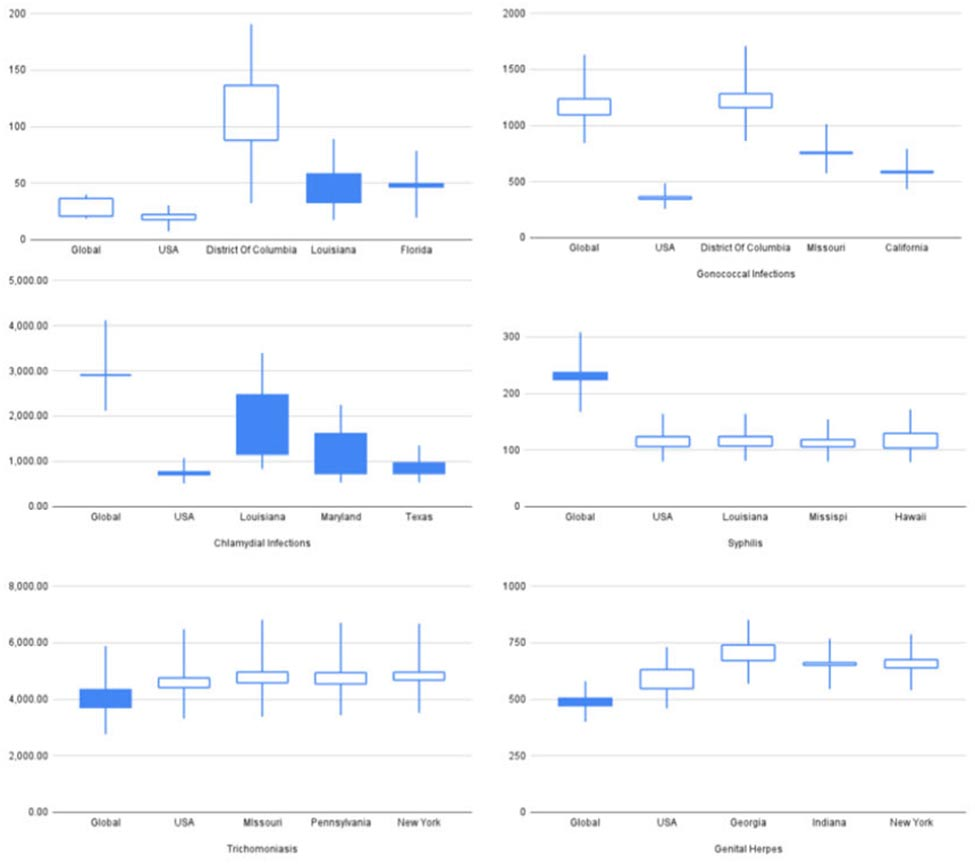
Boxplots comparing global, national (USA), and state-specific incidence rates of HIV and major STIs (chlamydia, gonorrhea, syphilis, trichomoniasis, genital herpes) by sex. Filled boxes indicate an increase in rates from 1990 to 2021; unfilled boxes indicate a decrease. Confidence intervals represent the combined range, using the highest upper bound and lowest lower bound from either 1990 or 2021 to capture maximum uncertainty. For each infection, global, national, and the top three U.S. states with the highest burden are displayed.

**Methods:**

We conducted a systematic, state-wise analysis for HIV and five major STIs from the Global Burden of Disease (GBD) 2021 data , comparing global, national (USA), and state-level trends. Analyses included age-standardized incidence by sex, state-wise comparisons, and risk factor attribution (unsafe sex, intimate partner violence), along with deaths and DALYs. Boxplots and choropleth maps visualized geographic and demographic disparities.

**Figure 3:**
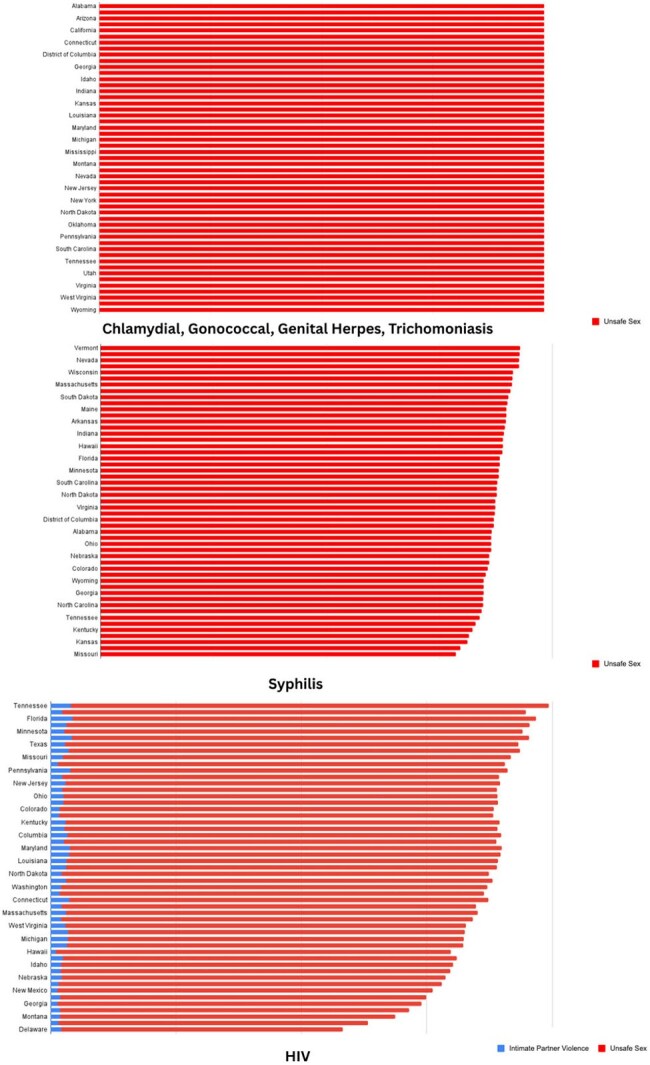
Bar graphs illustrating the proportion of STI burden attributable to unsafe sex and intimate partner violence by state showing the top three and lowest three incidence rates among states for HIV, chlamydia, and other key infections.

**Figure 4:**
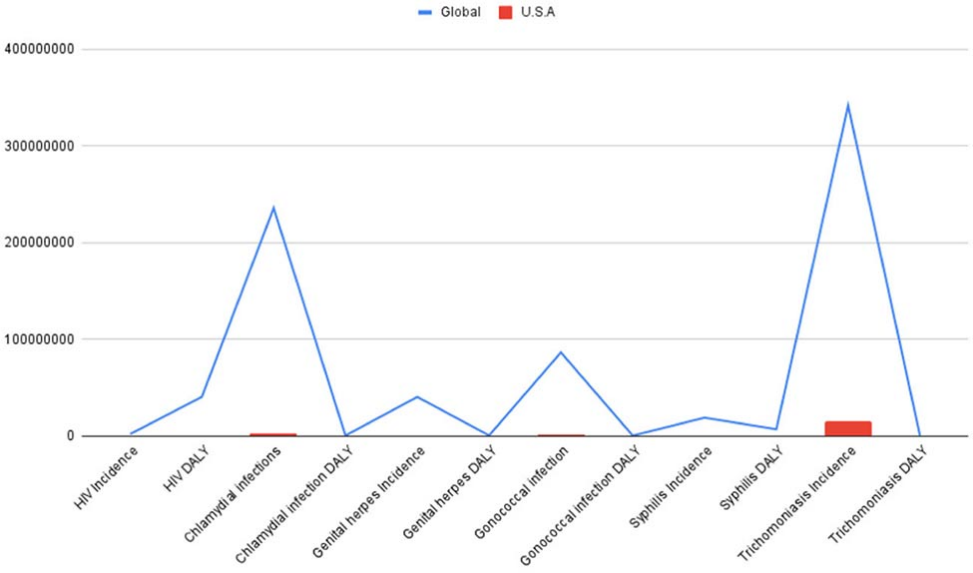
Gross incidence and DALY counts for HIV and major STIs in 2021, comparing global totals (blue line) with U.S. values (red bars). Global burdens far exceed national counts, with particularly high global DALYs for trichomoniasis and chlamydia.

**Results:**

Analysis revealed that while HIV incidence remains uniformly low across states, other STIs show substantial heterogeneity. The District of Columbia had the highest HIV incidence for both sexes; North Dakota and Vermont reported the lowest.Chlamydia peaked in Louisiana, with Minnesota and Nebraska at the lowest. Genital herpes incidence peaked in Georgia (females) and Washington (males), and was lowest in New Jersey and Florida. Gonorrhea was highest in D.C. (females) and Mississippi (males), with lowest rates in Massachusetts. Syphilis was most prevalent in Louisiana (males) and D.C. (females); lowest in South Dakota and Michigan. Trichomoniasis showed high female and overall incidence, peaking in Missouri, and lowest in California.Unsafe sex was the predominant risk factor for all STIs, with intimate partner violence contributing notably to HIV and syphilis in select states.

**Conclusion:**

Substantial geographic and sex-based disparities exist in the burden of HIV and STIs across US states. These patterns call for state-specific and gender-sensitive interventions, promoting safer sexual practices and enhancing screening initiatives. Targeted prevention and intervention strategies, particularly addressing unsafe sexual practices, are essential. These data-driven insights are pivotal for shaping targeted public health policies and resource allocation to mitigate the burden of STIs in the United States.

**Disclosures:**

All Authors: No reported disclosures

